# The clinical significance of single or double bands in cerebrospinal fluid isoelectric focusing. A retrospective study and systematic review

**DOI:** 10.1371/journal.pone.0215410

**Published:** 2019-04-15

**Authors:** Harald Hegen, Anne Zinganell, Michael Auer, Florian Deisenhammer

**Affiliations:** Department of Neurology, Medical University of Innsbruck, Innsbruck, Austria; Charite Universitatsmedizin Berlin, GERMANY

## Abstract

**Background:**

The presence of ≥3 oligoclonal bands (OCB) in the cerebrospinal fluid (CSF) without corresponding bands in serum represents a definite pathological pattern, whereas the clinical significance of 1–2 CSF bands (borderline pattern) is poorly investigated.

**Methods:**

We screened 1986 consecutive CSF and serum samples which were collected over a four-year time period and had results of isoelectric focusing (IEF) available. Of patients with borderline OCB we reviewed individual medical charts for assessment of clinical diagnoses. Where feasible, IEF was replicated and results of follow-up samples were obtained. IEF was performed using polyacrylamide gel followed by immunoblotting and IgG-specific antibody staining. Additionally, we performed a systematic literature review of the diagnostic specificity of OCB using different cut-offs for CSF-restricted bands.

**Results:**

Out of 253 patients with borderline OCB, 21.7% had an inflammatory neurological disease (IND) of the central nervous system, comprising 4% multiple sclerosis patients, and 14.2% had a peripheral IND, whereas the remaining 64.1% of patients showed non-inflammatory diseases. Frequency of one or two CSF bands without corresponding serum bands did not differ between the disease groups. In a subgroup of 100 patients IEF was repeated. Of those, 73% were OCB negative, while no sample was positive. In 26 patients IEF results were available of a follow-up sample collected after a median of 27 months. Of those, 4 (15.4%) turned positive. Systematic literature review revealed a diagnostic specificity of OCB of 97% and 92% using a cut-off ≥3 and ≥2 CSF bands in patients with mainly non-inflammatory neurological diseases.

**Conclusion:**

The clinical significance of one or two CSF-restricted bands is moderate and, hence, indicates a possible but not reliable proof of intrathecal B-cell activity. Sample re-testing, introduction of an additional diagnostic category, e.g. “possible intrathecal IgG synthesis”, and follow-up lumbar puncture might be possible options to address this scenario.

## Introduction

Detection of intrathecal IgG synthesis is part of the routine cerebrospinal fluid (CSF) work-up [1]. Isoelectric focusing (IEF) and subsequent immunoblotting is the gold standard to visualize clonally restricted IgG known as oligoclonal bands (OCB) in CSF [2]. Five different patterns of OCBs have been defined whereby the appearance of OCB in CSF without corresponding bands in serum constitute a local, intrathecal synthesis of IgG [3]. The presence of OCB in CSF supports the diagnosis of a variety of inflammatory central nervous system (CNS) diseases extending from an autoimmune to infectious pathology [1]. With regard to the cut-off defining OCB positivity, one might think that a consensus on ≥2 CSF bands has already been reached, as this threshold is recommended in the current MS diagnostic criteria [4] and suggested by recent review articles [5]. However, this cut-off is still equivocal. There is no explicit threshold recommended by CSF guidelines [1–3] and several studies have been published that support ≥3 CSF bands to define OCB positivity [6–9]. Also, when approaching this topic from a different perspective, there a few studies that investigated the clinical significance of a single CSF band [10,11], whereas the value of double CSF bands has not been addressed so far.

Therefore, we aimed i) to establish the frequency and disease associations of single or double CSF bands (borderline OCB pattern) in a large number of patients, ii) to perform IEF replication experiments in order to assess reliability and reproducibility of borderline OCB, iii) to determine OCB status in follow-up CSF samples. We also performed a systematic review of literature in order to determine diagnostic specificity of OCB in published cohorts of healthy subjects or patients with non-inflammatory neurological diseases considering different cut-offs for OCB positivity.

## Materials and methods

### Patients and samples

We have stored the results of CSF and serum sample analyses which were performed for routine diagnostic purposes from patients with mainly neurological diseases in a computerized database at the Neuroimmunology Laboratory of Medical University of Innsbruck–a reference laboratory for Western Austria. In a time period of four years IEF was carried out in 1986 paired CSF and serum samples and results were registered as negative, positive, or borderline. OCB negative status was defined as no bands in CSF and serum, or as bands in the CSF identical to those in serum. OCB positivity was defined as three or more bands in CSF without corresponding bands in serum. Borderline OCB pattern was defined as one or two bands in CSF without corresponding bands in serum.

**Fig 1:**
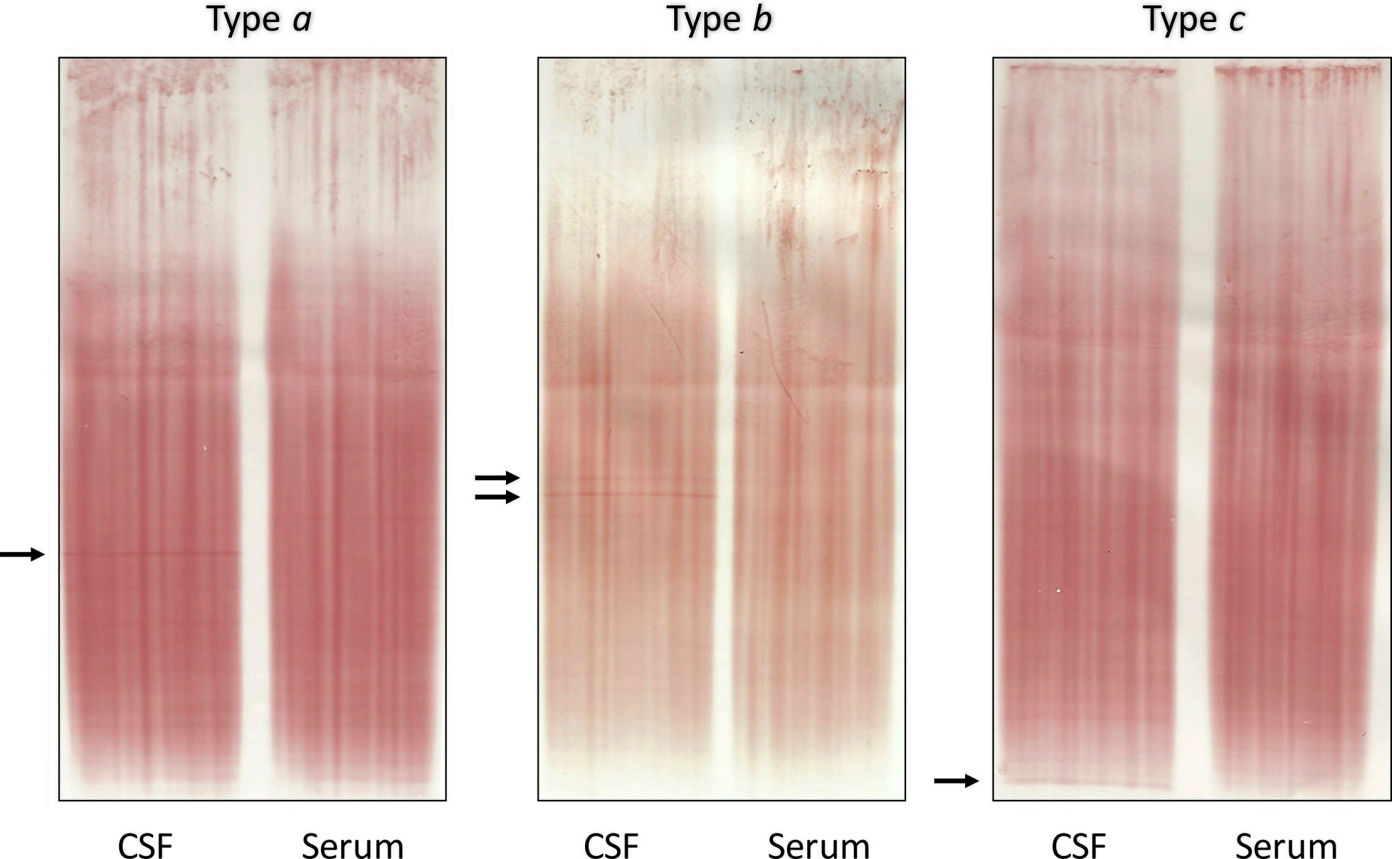
Borderline oligoclonal bands. Borderline OCB pattern was defined as one clear CSF band (type *a*) or two clear CSF bands (type *b*) without corresponding band(s) in serum, or as weak bands in CSF that are not clearly distinguishable from artefacts (type *c*). *Abbreviations*: CSF, cerebrospinal fluid; OCB, oligoclonal bands.

Results of borderline OCB pattern were reviewed by a blinded neurologist trained in OCB assessment (FD) and further stratified into three sub-patterns (Fig 1): one sharp band in CSF without corresponding band in serum (type *a*), two sharp bands in CSF without corresponding bands in serum (type *b*), and faint bands only in CSF that were not clearly distinguishable from artefacts (type *c*).

**Fig 2:**
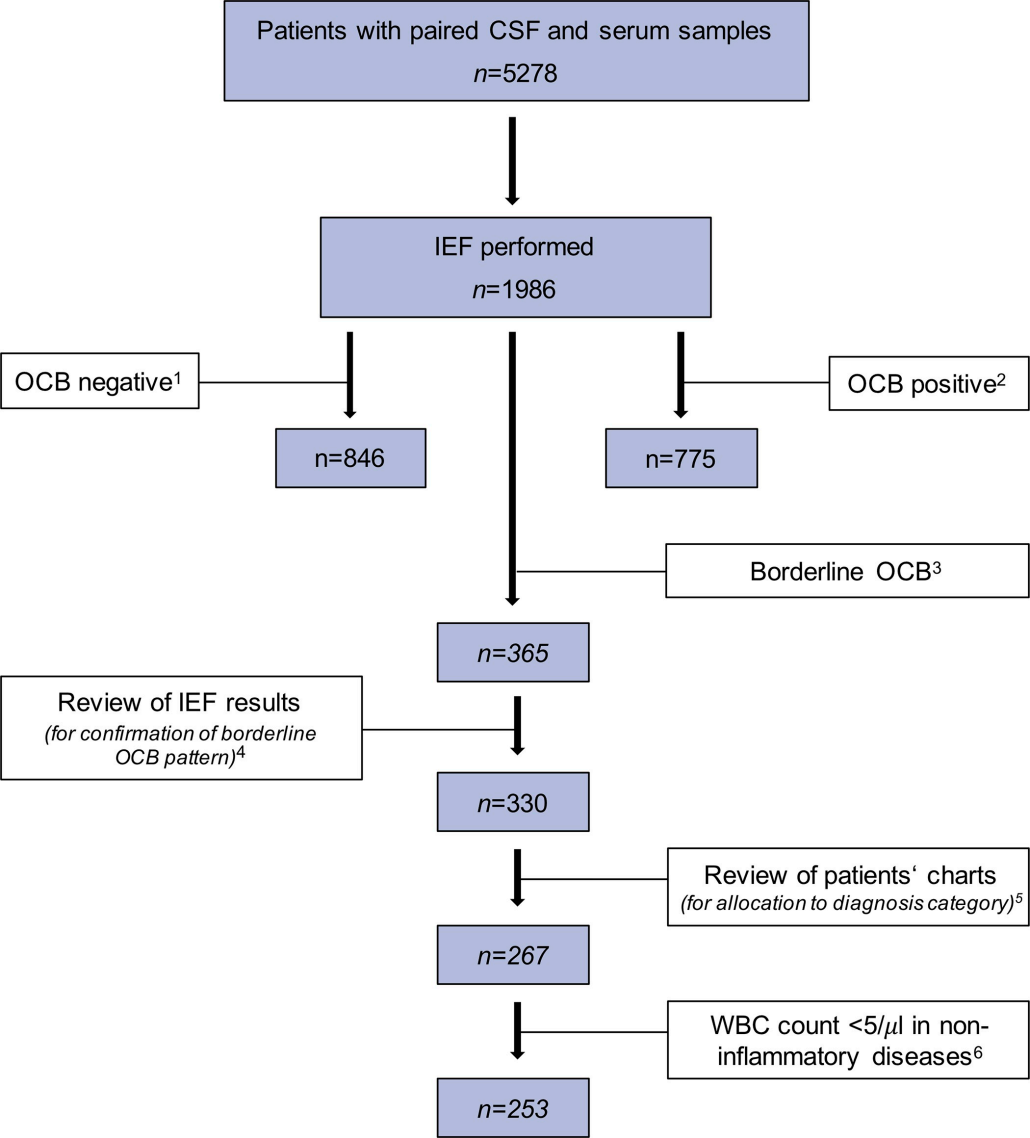
Sample flow chart. ^1^ OCB negative status was defined as no bands in CSF and serum, or as bands in the CSF identical to those in serum. ^2^ OCB positive status was defined as three or more bands in the CSF without corresponding bands in serum. ^3^ Borderline OCB pattern was the case when one or two clear bands were present in the CSF without corresponding band(s) in serum, or when weak bands in CSF were not clearly distinguishable from artefacts. ^4^ Original borderline OCB pattern could not be recovered in 35 CSF and serum sample pairs which were excluded from analysis. ^5^ A total of 63 patients were excluded because clinical information was either missing or insufficient for allocation to disease groups (according to Teunissen et al. Mult Scler. 2013 Nov;19(13):1802–9). ^6^ Patients with non-inflammatory diseases (i.e. of the NIND, SC, and no neurological disease group) require normal CSF WBC count. Therefore, a total of 10 patients of the NIND (two patients each with traumatic brain injury, stroke, CNS neoplasia, subcortical arteriosclerotic encephalopathy, polyneuropathy) and 4 of the SC group (three with vertebrogenic syndromes and one with headache associated with infection) showing a median WBC count of 6/μl were excluded. *Abbreviations*: CNS, central nervous system; CSF, cerebrospinal fluid; IEF, isoelectric focusing; NIND, non-inflammatory neurological disease; OCB, oligoclonal bands; SC, symptomatic control; WBC, white blood cell.

Individual medical charts of patients with borderline OCB were reviewed to obtain clinical diagnoses (Fig 2). When more than one neurological disease was evident in a single patient, the most CSF-relevant diagnosis was chosen (e.g. demyelinating or infectious CNS diseases rather than vascular and degenerative CNS diseases). Neurological diagnoses were eventually allocated to diagnostic groups according to the guidelines by the BioMS consortium (inflammatory neurological disease [IND], peripheral inflammatory neurological disease [PIND], non-inflammatory neurological disease [NIND], symptomatic control [SC]) [12] or labelled as no neurological disease [NND] as appropriate.

IEF was repeated in a subset of patients with available CSF and serum samples (n = 100). These samples were stored at -20°C until re-analysis. Furthermore, in another subset of patients (n = 26), IEF results of a follow-up sample were available.

### Isoelectric focusing and immunoblotting

Detection of OCB was performed by isoelectric focusing and subsequent immunoblotting using IgG-specific antibody staining as previously described by Keir et al. apart from using polyacrylamide instead of agarose gel [13].

15 μl of CSF and serum (diluted in Aqua dest. to achieve an IgG concentration of 3 mg/dl as appropriate) were applied to polyacrylamide gel (7.5%) covering a pH range of 3–10. Isoelectric focusing was carried out using 1N H_3_PO_4_ for the anode and 1N NaOH for the cathode. The samples were run for 2 hours (1.08 kV, 15 mA, 200W). After that, gels were mechanically blotted on nitrocellulose membranes over 20 minutes. Membranes were then placed in blocking solution (20 g/L dried, skimmed milk in 0.9% NaCl) for 30 minutes and washed three times with 0.9% NaCl. For immunolabelling, membranes were incubated for 1 hour with goat anti-human IgG (Cat. No. 2040–01, Southern Biotech, Birmingham, AL, USA) diluted 1:2000 in 50 ml diluent (2 g/L dried, skimmed milk in 0.9% NaCl). Rinsing with tap water for ten times was followed by one wash in diluent for 5 minutes. Thereafter, membranes were incubated with horseradish peroxidase-labelled rabbit anti-goat IgG (Ca. No. P0160, Agilent Dako, Santa Clara, CA, USA) diluted 1:1000 in 50 ml diluent for 1 hour. Another rinsing with tap water for ten times was followed by one wash in 0.9% NaCl for 5 minutes. Staining was performed by using 25 mg of 3-amino-9-ethylcarbazole diluted in 10 ml ethanol and 50 ml acetate buffer. After adding 50 μl of 30% hydrogen peroxide, membranes were incubated for 15 minutes. After development of the red-brown bands, membranes were washed with distilled water and air-dried.

### Literature search

**Fig 3:**
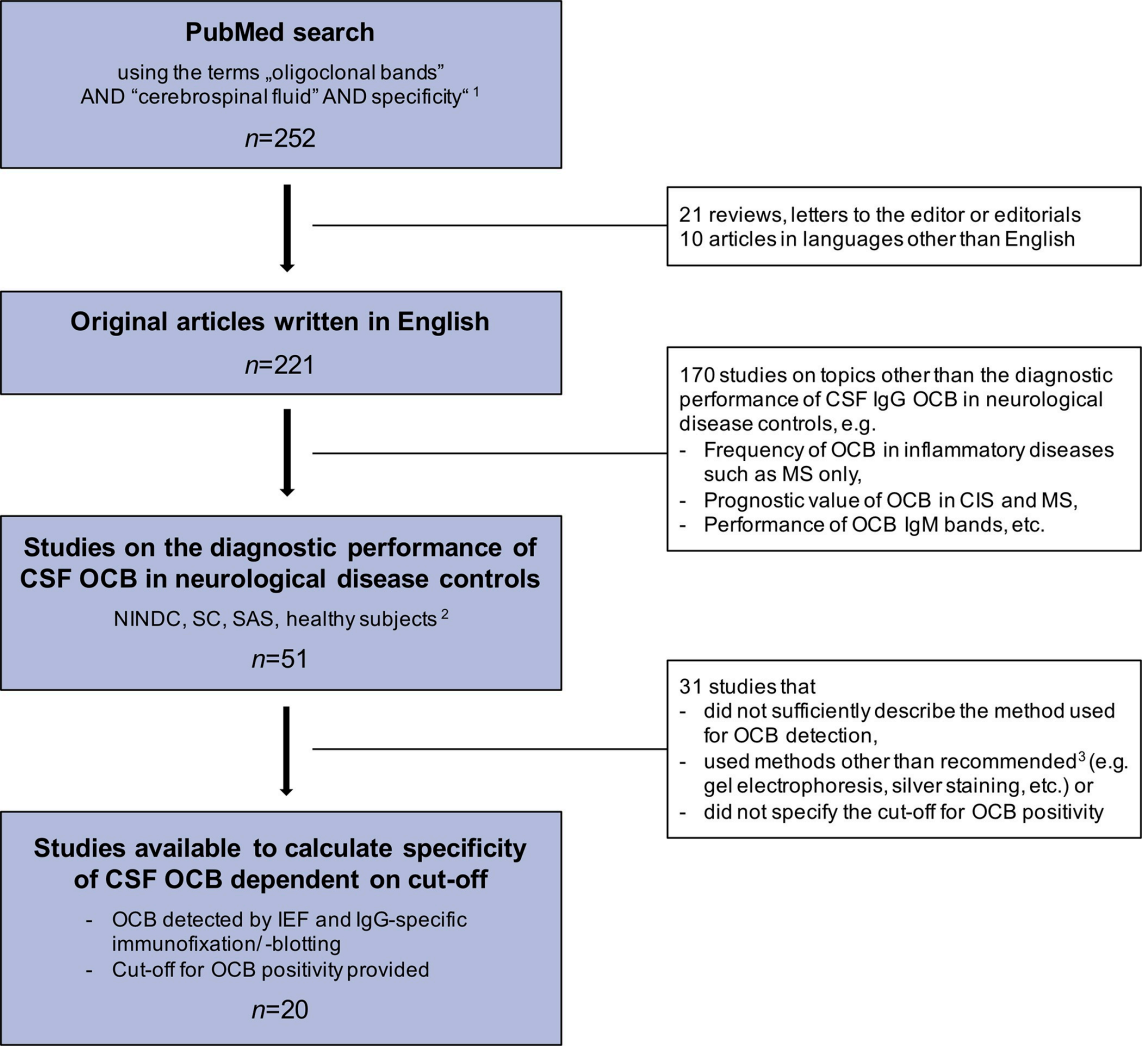
Literature search criteria. ^1^ limited to 1^st^ September 2018 ^2^ Studies were eligible when NINDC, SC, SAS or healthy subjects (Teunissen et al. Mult Scler. 2013 Nov;19(13):1802–9) were part of study population. ^3^ Freedman et al. Arch Neurol. 2005 Jun;62(6):865–70; Andersson et al. J Neurol Neurosurg Psychiatry. 1994 Aug;57(8):897–902 *n*, number of items returned by PubMed search *Abbreviations*: CIS, clinically isolated syndrome; CSF, cerebrospinal fluid; IEF, isoelectric focusing; IgG, immunoglobulin G; IgM, immunoglobulin M; MS, multiple sclerosis; NINDC, non-inflammatory neurological disease controls; OCB, oligoclonal bands; SAS, spinal anesthesia subjects; SC, symptomatic controls.

A literature search in PubMed using the search terms “oligoclonal bands” AND “cerebrospinal fluid” AND “specificity” limited to 1^st^ September 2018 was performed and returned 252 references. All abstracts were screened and only original articles written in English were considered. Abstracts that primarily did not deal with the diagnostic performance of OCB in the CSF of neurological disease controls were excluded. Only studies were considered which detected OCB by means of IEF followed by IgG-specific immunofixation/ -blotting, specified a cut-off for positive CSF OCB, provided patients’ clinical diagnoses allowing allocation to disease categories and presented results for diagnostic specificity (main aim; Fig 3). If available, diagnostic sensitivity of MS patients was retrieved as well.

Out of the selected studies, the following data were extracted and inserted into a piloted form: type of gel used for IEF, application of immunoblotting or–fixation, total number of patients, applied cut-off for CSF-restricted bands to define OCB positivity, number of control subjects, characteristics of control groups allowing assignment to one of the following categories: SC, NIND, PIND, IND, spinal anesthesia subjects (SAS) [12] or healthy controls, number of MS patients if applicable, number of OCB positive and OCB negative patients, diagnostic specificity and sensitivity. For control groups only non-inflammatory conditions (NIND, SC, SAS and healthy subjects) were considered. In cases, where results of OCB positivity were not separately shown for non-inflammatory and inflammatory diseases (IND, PIND) the overall findings were used and a “best” and “worst” case scenario for the diagnostic performance of OCB was calculated. A review protocol does not exist.

### Statistical analysis

Statistical analysis was performed using SPSS 25.0 (SPSS Inc., Chicago, IL, USA). Frequency distributions were analysed by χ^2^ test. Comparisons of non-parametric data such as IgG index between groups was performed by Kruskal-Wallis or Mann-Whitney U test. A p-value <0.05 was considered statistically significant.

With regard to the systematic review of studies, results of studies using the same cut-off to define OCB positivity (i.e. either ≥2 and ≥3 CSF-restricted bands) were combined in order to calculate diagnostic sensitivity and specificity.

### Ethics

No ethical vote was needed because this is an anonymous retrospective analysis of existing data that were obtained in routine diagnostic procedures and used for quality assurance purposes within this study.

## Results

### Clinical diagnoses in borderline OCB pattern

Out of 1986 CSF and serum sample pairs, 330 (16.6%) showed borderline OCB pattern. Samples were excluded, when available clinical information was not sufficient to allocate patients to a disease group (IND, PIND, NIND, SC or NND) or when particular requirements defining non-inflammatory neurological diseases, i.e. CSF WBC count, were not met. This resulted in a total of 253 patients with borderline OCB pattern who were finally available for statistical analysis (Fig 2).

Of this cohort, median age was 45.3 years (5^th^-95^th^ percentile: 18.9–75.1) with a balanced sex ratio (49.8% females). Overall, 55 (21.7%) patients showed IND, whereas 36 (14.2%) were classified as PIND, 89 (35.2%) as NIND, 52 (20.6%) as SC and 21 (8.3%) as NND group. Demographic and main CSF findings of each disease group are shown in Table 1. Detailed clinical diagnoses of patients are listed in S1 Table. The sub-pattern *a* occurred in 81 (32.0%), *b* in 41 (16.2%) and *c* in 131 (51.8%) of cases. Frequency of sub-pattern did not differ between disease groups (p = 0.365; S2 Table). The routine CSF analytes (WBC count, CSF total protein, Q_alb_, IgG index and frequency of patients with positive IgG_IF_ or RBC count >500/μl) were similar across borderline OCB sub-patterns.

**Table 1:** Demographic and routine CSF findings according to disease groups.

	IND	NIND	PIND	SC	NND
**n**	55	89	36	52	21
**Age**, years	39.3 (16.7–75.1)	51.7 (21.6–76.3)	57.5 (26.8–77.0)	36.8 (22.3–67.9)	37.6 (22.3–57.4)
**Sex**, female, n (%)	24 (43.6)	40 (44.9)	12 (33.3)	35 (67.3)	15 (71.4)
**RBC**, /μl	3 (0–4640)	1 (0–1600)	1 (0–4800)	0 (0–250)	0 (0–3360)
**WBC**, /μl	11 (0–1600)	1 (0–3)	1 (0–10)	1 (0–3)	0 (0–3)
**CSF total protein**, mg/dl	65 (30–291)	52 (30–93)	81 (44–236)	47 (24–75)	42 (22–70)
**Q**_**alb**_	9.4 (3.5–49.4)	6.5 (3.3–15.4)	12.5 (4.8–45.9)	5.7 (3.0–10.3)	4.7 (2.3–11.6)
**IgG index**	0.53 (0.39–0.79)	0.47 (0.39–0.60)	0.54 (0.40–0.76)	0.49 (0.38–0.61)	0.49 (0.36–0.65)
**IgG**_**IF**_, n (%)	1 (2)^#^	0	0	0	0

Data are shown as median (5^th^– 95^th^ percentile) unless specified otherwise. ^#^ % IgG_IF_ according to Reiber (Reiber H. J Neurol Sci. 1994;122:189–203) and Auer & Hegen (Auer M, Hegen H et al. Eur J Neurol. 2016;23:713–721) formulae was 62% and 66%, respectively, in the single patient of the IND group. Clinical diagnosis of this patient was HIV encephalopathy.

*Abbreviations*: CSF, cerebrospinal fluid; HIV, human immunodeficiency virus; IF, intrathecal fraction; IgG, immunoglobulin G; IND, inflammatory neurological disease; NIND, non-inflammatory neurological disease; NND, no neurological disease; PIND, peripheral inflammatory neurological disease; Q_alb_, CSF/ serum albumin quotient; RBC, red blood cell; SC, symptomatic control; WBC, white blood cell

### Reproducibility of borderline OCB pattern

**Fig 4:**
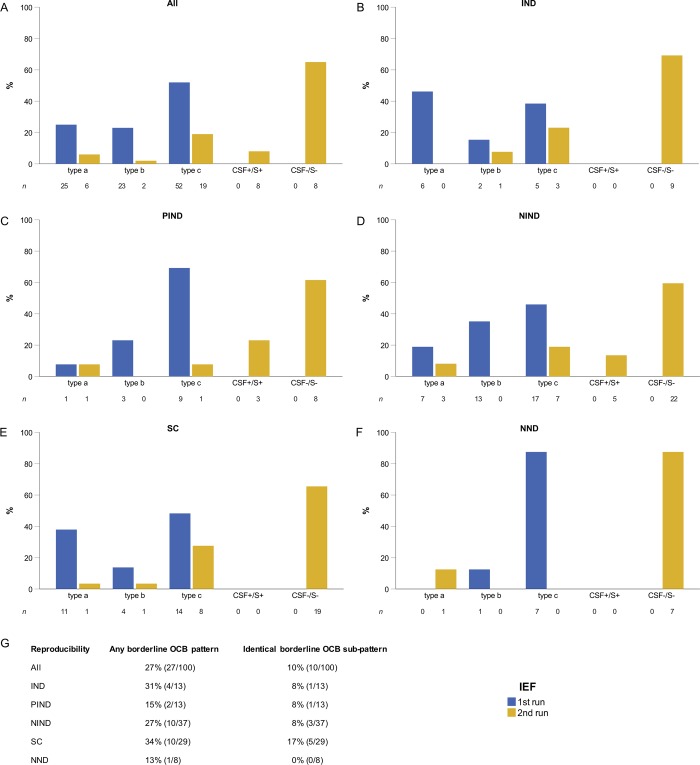
Reproducibility of borderline OCB pattern. Results in a subgroup of patients (n = 100) eligible for IEF replication experiments are shown. The 1^st^ run of IEF shows the frequency of original OCB sub-pattern *a*, b and *c*, whereas the 2^nd^ run of IEF shows the results of the replication experiments. Results are shown as percentage for (A) the whole patient group, (B) patients with IND, (C) PIND, (D) NIND, (E) SC and (F) NND. In panel (G) reproducibility of any borderline OCB pattern as well as of OCB sub-pattern are shown. *Abbreviations*: CSF+/S+, OCB in the CSF with identical bands in serum; CSF-/S-, no bands in CSF and serum; IEF, isoelectric focusing; IND, inflammatory neurological disease; NIND, non-inflammatory neurological disease; NND, no neurological disease; OCB, oligoclonal bands; PIND, peripheral inflammatory neurological disease; SC, symptomatic control.

A total of 100 CSF and serum sample pairs were re-analysed by IEF. In those, OCB were negative in 73 samples (65 with no OCB, and 8 with identical bands in CSF and serum), whereas in 27 samples a borderline OCB pattern was reproduced. In the latter case, an identical sub-pattern occurred in 10 and a different sub-pattern in 17 samples. No borderline OCB pattern tested positive OCB when IEF was repeated. Distribution of the sub-patterns (type *a*, *b* and *c*) did not significantly change between original and re-IEF (p = 0.137), however, there was a significant shift to OCB negative status (p<0.001; Fig 4).

In order to identify factors that might have influenced reproducibility of borderline OCB pattern, we addressed the following issues. First, we investigated whether a certain disease group (e.g. IND) was associated with reproducibility of OCB. The frequency of reproducible borderline OCB pattern was similar between the disease groups (p = 0.616; Fig 4) and did not differ when patients with IND were compared to the remaining patients (30.8% in the IND group vs. 26.4% in the “non-IND”; p = 0.743). Second, median IgG index did not significantly differ between samples which were classified as OCB negative and OCB borderline by the re-IEF (0.50 vs. 0.51, p = 0.907; for further details see S3 Table). Third, we looked at the influence of CSF storage time on the reproducibility but did not observe a significant difference in storage time between samples with reproducible OCB pattern and those without (median 20.2 vs. 22.2 months; p = 0.768).

### Follow-up isoelectric focusing

In 26 patients, results from a follow-up IEF were available. Six (23.1%) patients belonged to the IND group, whereas 3 (11.5%) were classified as PIND, 12 (46.2%) as NIND, 2 (7.7%) as SC and 3 (11.5%) patients as NND. In total, follow-up samples of 4 (15.4%) patients tested OCB positive, 9 (34.6%) showed again borderline OCB pattern, and 13 (50%) were OCB negative (7 with no bands, and 6 with identical bands in CSF and serum). The median time interval between baseline and the follow-up IEF was overall 27 months; in the OCB positive group 33 months as opposed to 22.5 months in the remaining patients. Two of the four patients who turned OCB positive at follow-up belonged to the IND group (one patient with MS, one patient with myelitis; baseline OCB sub-pattern in both cases was type *c*;), the other two patients belonged to the NIND group (type *b* at baseline).

### Literature search

We identified 20 studies fulfilling our search criteria. A total of 17 studies using a cut-off of ≥2 CSF bands included 3451 patients with predominantly non-inflammatory diseases (i.e. in >97% of cases). Four studies applying a cut-off of ≥3 CSF bands comprised 1002 patients with solely non-inflammatory diseases. Combined evaluation of these studies revealed a diagnostic specificity of 92% using a cut-off of ≥2 CSF bands, whereas specificity was 97% by a cut-off of ≥3 CSF bands. Diagnostic sensitivity for MS was 87% and 90% applying a cut-off of ≥2 and ≥3 CSF bands, respectively. The main findings are summarized in Table 2. Details on the calculation of diagnostic specificity are displayed in S4 Table and S5 Table.

**Table 2:** Reports on the diagnostic value of oligoclonal bands.

Publication	Type of gel	Immunoblotting/ -fixation	Number of patients	CSF bands cut-off	Disease groups	Control groups–Categories	Control groups –Details	Oligoclonal bands	
								Sensitivity	Specificity
Bayart 2018	agarose	immunoblotting	98	≥2	MS	NIND	according to BioMS guidelines	46/59 (78%)	39/39 (100%)
Christiansen 2018	agarose	immunofixation	193	≥2	MS	SC NIND PIND IND	SC: 66 patients with paresthesia, visual disturbances or vertigo, but no objective neurological deficits or MRI findings suggestive of MS (except for one RIS patient) NIND: 28 patients with PNP, spastic paraplegia, stroke, facial nerve palsy, headache, cerebellar ataxia, essential tremor, motor neuron disease, myotonic dystrophy, TBI, vestibular neuritis, intracranial hypertension. PIND: 2 patients with GBS IND: 1 patient with meningoencephalitis	79/96 (82%)	91/97 (94%)
Gurtner 2018	agarose	immunoblotting	211	≥2	MS^1^	NIND	Patients with degenerative, non-inflammatory or peripheral neurological diseases and cancer	63*/67 (94%)	121*/144 (84%)
				≥3				59*/67 (88%)	127*/144 (88%)
				≥4				58*/67 (87%)	130*/144 (90%)
Dias-Carneiro 2016	agarose	immunoblotting	58	≥2	MS	SC NIND	SC: 15 patients with primary headaches NIND: 2 patients with compressive myelopathies, 1 each with spinal tumor, conversion disorder, ischemic optic neuropathy	23/32 (72%)	25/26 (96%)
Hegen 2016	PAGE	immunoblotting	161	≥3	n.a.	SC	according to BioMS guidelines	n.a.	161/161 (100%)
Zeman 2015	agarose	immunofixation	122	≥2	MS	NIND SC	NIND: 79 patients with migraine, PNP, vertebrogenic disease, radiculopathy, CNS tumor, vertigo, ischaemic stroke, IFNP, motor neuron disease, dementia. SC: 15 patients with no specific neurological disorder (mainly mild mood and/or psychosomatic disorders)	23/28 (82%)	83/94 (88%)
Abraira 2011	agarose	immunofixation	81	≥2	MS	NIND	Patients with stroke, NPH, ALS, paraneoplastic syndrome, intracranial hypertension, hereditary spastic paraplegia, epilepsy, Lewy body disease, migraine, pineal germ cell tumor	49/52 (94%)	28/29 (97%)
Gama 2009	PAGE	immunoblotting	109	≥2	MS	SAS	-	49*/90 (54%)	19*/19 (100%)
Mygland 2007	agarose	immunoblotting	269	≥2	MS	SC NIND	SC: 142 patients with symptoms but without proven neurological cause 113 patients with NIND	13/14 (93%)	245/255 (96%)
Sa 2005	n.s.	immunoblotting	242	≥2	MS	NIND	The three most frequent diagnoses were ischaemic stroke, neurodegenerative disorder and spondilotic myelopathy	58/69 (84%)	167/173 (97%)
Bednarova 2005	agarose	immunoblotting	57	≥2	MS	NIND IND	NIND: 8 patients with IFNP, vertebrogenic disorders, neurasthenia, polyneuropathy and neurodegenerative disorder. IND: 7 patients with bacterial meningitis, aseptic meningitis and sepsis.	34*/42 (81%)	14*/15 (93%)
Villar 2005	agarose	immunoblotting	466	≥2	MS	NIND SC IND PIND	100 patients with NIND SC: 39 patients with nonspecific headaches without any neurologic abnormalities 37 patients with IND different from MS and CNS infectious diseases: myelitis, CNS vasculitis, neurolupus, paraneoplastic syndrome, Behçet disease, Rasmussen disease, Hashimoto encephalitis, gluten ataxia, neurosarcoidosis, Sjögren disease. 26 with PIND	127/132 (96%)	332*/334 (99%)
Fortini 2003	agarose	immunoblotting	71	≥4	MS	NIND	Patients with PNP, dementia, hereditary spastic paraparesis, epilepsy, spinocerebellar ataxia and transient ischemic attack	18*/20 (90%)	48*/51 (94%)
Haghighi 2000	PAGE	immunoblotting	97	≥2	MS	Healthy	-	45/47 (96%)	48/50 (96%)
Marchetti 1999	agarose	immunoblotting	43	≥2	MS^2^	NIND	Patients with non-inflammatory CNS diseases	20/21 (95%)	20/22 (91%)
Cowdrey 1993	PAGE	immunoblotting	166	≥2	MS	NIND IND PIND	NIND: 133 Patients with headache, trauma, skeletal disorders degenerative and movement diseases, etc. IND: 7 patients with e.g. meningitis PIND: 2 patients with GBS and CIDP *For details see publication*^3^	21/22 (95%)	142/144 (99%)
Öhman 1992	agarose	immunoblotting	323	≥2	MS	NIND	Patients not to have any neurological disease affecting the CNS, e.g. tension headache, uncharacteristic dizziness, and mild psychoneurotic disorders.	104/112 (93%)	207/211 (98%)
				≥3	MS	NIND		100*/112 (89%)	207/211 (98%)
McLean 1990	agarose	immunoblotting	692	≥3	MS	NIND	Patients with headache; skeletal, vascular, degenerative, psychiatric, neoplastic, toxic, paroxysmal, metabolic, congenital or systemic disorders; neuromyopathie, trauma^§^	186/206 (90%)	477/486 (98%)
Kostulas 1987	agarose	immunoblotting	955	≥2	MS	NIND	Patients with IFNP, headache, epilepsy, cerebrovascular disease, dementia Parkinson’s disease, radicular syndrome, PNP, mononeuropathy, psychoneurosis, paresthesia, myelopathy, CNS tumor, trigeminal damage, vertigo^§^	58/58 (100%)	813/897 (91%)
Link 1983	agarose	immunoblotting	949	≥2	MS	NIND PIND	NIND: Patients with Parkinson’s disease, ALS, CNS tumor, PNP, intracranial haemorrhage, dementia, stroke, etc.^§^ PIND: 14 patients with GBS *For details see publication*	41/43 (95%)	780/902 (86%)

Only studies were considered which detected OCB by means of IEF followed by IgG-specific immunofixation/ -blotting, specified a cut-off for CSF OCB used to calculate diagnostic sensitivity and specificity and provided patients’ clinical diagnoses allowing allocation to disease categories.

^1^ comprised patients predominantly with MS (n = 62), but also with CIS (n = 3) and RIS (n = 2).

^2^ comprised also patients with probable MS according to Poser criteria (n = 16).

^3^ The diagnoses of two control patients were not specified in the original publication.

^§^ Patients with non-inflammatory diseases were grouped to calculate diagnostic specificity of OCB.

* Number of OCB positive/ negative patients was not specified in the original publications, but calculated using % of OCB positive/ negative patients and total number of patients.

*Abbreviations*: ALS, amyotrophic lateral sclerosis; CIDP, chronic inflammatory demyelinating polyneuropathy; CIS, clinically isolated syndrome; CNS, central nervous system; GBS, Guillain-Barré syndrome; IFNP, idiopathic facial nerve palsy; IND, inflammatory neurological disease; MS, multiple sclerosis; n.a., not appropriate; NIND, non-inflammatory neurological disease; NPH, normal pressure hydrocephalus; n.s., not specified; OCB, oligoclonal bands; PAGE, polyacrylamide gel; PIND, peripheral inflammatory neurological disease; PNP, polyneuropathy; RIS, radiologically isolated syndrome; SAS, spinal anesthesia subjects; SC, symptomatic control, TBI, traumatic brain injury

## Discussion

The detection of OCB in CSF is the gold standard to prove intrathecal IgG synthesis indicating sustained inflammation within the CNS compartment supporting the diagnosis of a variety of neurological diseases, for example multiple sclerosis (MS) [1]. However, the cut-off defining OCB positivity, that is the number of bands in the CSF without corresponding bands in serum, is still not unquestioned.

The majority of studies on the diagnostic accuracy of OCB (including those cited by published CSF guidelines [2]) used a cut-off of ≥2 [7,14,15] or ≥3 CSF bands [7,8]. Besides, a few studies exist which addressed the clinical significance of a single CSF band as obtained by IEF and subsequent immunoblotting [10,11,16], while the significance of double CSF bands has not been investigated so far. Hence, for our analysis we included patients with one or two CSF bands. We aimed to investigate the clinical relevance of this borderline OCB pattern in a three-step process.

First, we reviewed clinical diagnoses of patients with borderline OCB. We observed that 78% of samples were assigned to diseases other than inflammatory CNS diseases, while an IND was found in only 22% of cases. Furthermore, IND did not occur more frequently in one of the OCB sub-pattern, i.e. the diagnostic value of one or two CSF band(s) was similar. A closer look on the clinical diagnoses within the IND group reveals that about half of patients suffered from chronic inflammatory diseases, which are typically associated with intrathecal IgG synthesis (e.g. MS), whereas the remaining showed predominantly acute inflammatory disorders which are not necessarily associated with OCB (e.g. meningitis).

Second, we performed replication experiments in a subgroup of patients which revealed that the majority of samples were OCB negative when re-tested. In contrast, borderline OCB pattern was reproduced in only 27%, exactly the same sub-pattern in 10% of patients. To consider various factors that might have influenced reproducibility of IEF results, we compared the frequency of disease groups, CSF storage time and amount of (intrathecal) IgG between samples with reproducible and non-reproducible borderline OCB pattern in the second run and did not find any significant differences. It is not clear how this low reproducibility can be explained. Generally, most IEF results are unambiguous, i.e. either negative–without any CSF bands–or definitely positive–with numerous bands in the CSF. Miscounting e.g. one of ten bands results in a small relative error without changing the overall positive OCB result whereas miscounting one of few bands has a larger relative effect and might lead to a false positive or negative OCB result. Previous studies investigating inter-observer agreement revealed good agreement with respect to the presence of OCB, but only poor agreement with respect to number of CSF bands [17,18] including counting of few CSF bands [19]. These factors explain to some extent why reproducibility of borderline OCB pattern was low in the present study. From a methodological point of view, especially with regard to subpattern *c* contributing approximately 50% of borderline OCB, one might argue that non-linearity of the pH gradient used for IEF is the cause [3]. Also, the possible presence of minimal physiological microheterogeneity of IgG was supposed to result in weak bands [17]. Irregularities in the surface of the gel or during immunoblotting steps might be an issue that could explain “loss” of CSF bands in the replication experiments. Other considerations such as too short IEF time resulting in diffuse bands because IgG have not reached the isoelectric point, or too long IEF time leading to gradient drift are very unlikely, as we adhered to a well-established IEF protocol [13] which did not differ between the first and second IEF run. The low reproducibility of one or two CSF bands could as well be the result of low amounts of clonally restricted IgG close to the detection limit of IEF.

Third, we were able to observe in a small subgroup of patients whether OCB status changed over time. Out of 26 patients, who had another lumbar puncture after a median time of 27 months, 4 (15.4%) turned OCB positive. This observation confirms a previous small study showing that out of 27 patients with a single CSF band, only a minority (33%) developed OCB positivity (defined as ≥2 CSF bands) during follow-up (of the remaining patients, 13 showed persistent single band and five were tested negative) [10]. There are further reports on the significance of a single CSF band [20], however, interpretation of results is difficult as these studies were conducted with agarose gel electrophoresis (instead of IEF) which is of low resolution and sensitivity [21].

There are several limitations of the study. Due to the retrospective nature of the study inclusion of patients depended on availability of clinical data, and selection of samples for replication experiments was based on availability of samples. Furthermore, follow-up samples were collected on a clinical routine basis and thus, were only available in a small subgroup of patients. A potential bias due to CSF storage and additional thawing cycle on reproducibility of borderline OCB cannot be ruled out, even though we did not observe a difference between storage time of samples with reproducible and non-reproducible OCB patterns. It has been shown that IgG are stable even in case of long-term storage (in these scenarios determined by immunoassays such as turbidi- or nephelometric methods; and stored at -25°C) [22], but the reproducibility of OCB after a longer time of storage has not been addressed so far. Also, the impact of different storage temperatures (-80°C vs. -20°C) on OCB recovery is not clear. This means it cannot be ruled out that the low reproducibility of less than a third of borderline OCB is at least to some extent due to the 20 months storage time and/ or only -20°C storage temperature of CSF and serum samples. Another issue is blood contamination due to traumatic lumbar puncture which might lead to false negative OCB in the CSF (especially in cases of low intrathecal IgG synthesis). However, the CSF samples showed a median RBC count of 1/μl and only 7% of patients had a RBC count >500/μl [23].

We showed that not only a single CSF band, as reported previously [10], but also two CSF bands do not reliably indicate intrathecal IgG synthesis as there was no compelling association with (chronic) inflammatory neurological diseases. The positive predictive value of 1–2 CSF bands to identify an inflammatory CNS disease reached only about 20%. Therefore, our results support a higher than the frequently used cut-off of ≥2 CSF bands to define OCB positivity. It is obvious that an increase in diagnostic specificity comes at the expense of diagnostic sensitivity as recently shown by a study applying different cut-offs ranging from ≥2 to ≥4 CSF bands [6]. However, we think that the advantage of increased specificity by excluding irrelevant diagnoses in the vast majority of cases overcomes the disadvantage of lower sensitivity. Surveying current evidence a cut-off of ≥3 CSF bands resulted in a specificity of 97% [6–8,24], i.e. allows less than 5% false positive results which is an accepted, tolerable rate in laboratory diagnostics [25], whereas a cut-off of ≥2 CSF bands resulted in a too low specificity of only 92% [6,7,14,15,21,26–38] (Table 2). At the same time, the diagnostic sensitivity of approximately 90% was similar when using a cut-off of ≥2 or ≥3 CSF bands [6–8,14,15,21,24,26–38]. One study determined ≥3 CSF bands by receiver operating characteristics as the best cut-off to discriminate between patients with MS and other neurological diseases, consisting predominantly of NIND [9].

At this point we also want to state that we detected OCB by IEF on polyacrylamide gel followed by immunoblotting and–labelling (as described above). Even when adhering to the recommendations on how to perform OCB detection [2,3], several technical differences usually remain between laboratories. It is known that e.g. different gels (e.g. agarose or polyacrylamide gel; different gel concentrations), different sample application, different applied sample volume at different IgG concentration, different parameters for carrying out IEF (in terms of applied voltage or duration) or different detection methods (e.g. labelling of the detection antibody with e.g. alkaline phosphatase or horseradish peroxidase) might impact on the sensitivity to detect OCB. And test sensitivity has an impact when determining cut-off values. This means that extrapolating the findings of our study needs to take this methodological variability into account. The same methodological limitations of OCB detection have to be considered in context with the results of our literature review. The presented specificities using a cut-off ≥2 or ≥3 CSF bands were calculated irrespective of the exactly used method to determine OCBs. This is certainly a limitation of the systematic review. Another limitation of the systematic review is that it is based on studies using patient cohorts that were largely compiled at the authors’ discretion and not recruited systematically. This bears the risk of bias in reported OCB frequencies.

Apart from cut-off considerations, borderline OCB might already indicate intrathecal IgG synthesis in some individual cases. Borderline OCB may be connected to the maturation of the humoral immune response. From the initial production of IgG with a wide range of affinity, this response leads to the selection of a few plasma cell clones that secrete high-affinity IgG, i.e. oligoclonal IgG [39]. This process requires time and explains why a certain proportion of patients converts from borderline OCB to full OCB positivity at follow-up [10,11]. These considerations can be the basis for repeating lumbar puncture in certain patients after some time although this constellation is rare. The overall frequency of single or double CSF band(s) was approximately 5% and 2.5%, respectively. Restricted to reproducible CSF bands, this number decreased to approximately 1%, that is in line with previous reports on CSF borderline pattern [10,11,40].

Altogether, we suggest rethinking the currently widely used cut-off of ≥2 CSF bands, as the clinical significance of 1–2 CSF bands is moderate and current evidence (Table 2) shows that diagnostic specificity is higher when using ≥3 CSF bands, i.e. >95%. We want to underline that 1–2 CSF bands might indicate an early intrathecal IgG synthesis. Repeating IEF can be considered in these borderline OCB pattern, so that a methodological and rater-dependent error is minimized. Furthermore, as suggested previously [39], we think that an additional category such as “possible intrathecal IgG synthesis” might be useful, as well as another lumbar puncture at follow-up in certain clinical constellations. Due to the methodological considerations made above and the lack of a real standardization of OCB detection, there is a need for a blinded, multicenter study that compares the various, currently used laboratory methods for OCB detection with respect to different OCB cut-offs.

## Supporting information

S1 Checklist PRISMA checklist. (PDF)

S1 Table Clinical diagnoses of all patients with borderline OCB pattern. AIDS, acquired immune deficiency syndrome; CNS, central nervous system; St.p., status post.
(PDF)

S2 Table OCB sub-pattern of all patients according to disease groups. In the whole cohort (n = 253), frequency of original OCB sub-pattern *a*, *b* and *c* are shown for each disease group (sub-pattern with the highest frequency are marked bold). *Abbreviations*: IND, inflammatory neurological disease; NIND, non-inflammatory neurological disease; NND, no neurological disease; OCB, oligoclonal bands; PIND, peripheral inflammatory neurological disease; SC, symptomatic control.
(PDF)

S3 Table Association of IgG index with reproducibility of borderline OCB pattern. Quartiles of IgG index were separated by values of 0.44, 0.50 and 0.56. IgG index was calculated as (CSF IgG/ serum IgG)/ (CSF albumin/ serum albumin). *Abbreviations*: IEF, isoelectric focusing; IgG, immunoglobulin G; OCB, oligoclonal bands; Q, quartile.
(PDF)

S4 Table Calculation of OCB diagnostic specificity using a cut-off ≥2 CSF-restricted bands. (1) Studies that included only patients with NIND and SC are shown and used for specificity calculation. (2) Studies that included patients with NIND/SC and IND/PIND are shown. The N of patients with IND/PIND are substracted from the total N of controls, but not from the N of OCB negative patients. This approach results in a "Best OCB" scenario, where excluded IND/PIND are considered OCB positive. (3) Studies that included patients with NIND/SC and IND/PIND are shown. The N of patients with IND/PIND are substracted from the total N of controls and from the N of OCB negative patients. This approach results in a "Worst OCB" scenario, where excluded IND/PIND are considered OCB negative. (4) Studies from (1) and (2) are combined. (5) Studies from (1) and (3) are combined. *Abbrevations*: IND, inflammatory neurological disease; N, number; OCB, oligoclonal bands; PIND, peripheral inflammatory neurological diseae; SC, symptomatic control.
(PDF)

S5 Table Calculation of OCB diagnostic specificity using a cut-off ≥3 CSF-restricted bands. *Abbreviations*: CSF, cerebrospinal fluid; N, number; OCB, oligoclonal bands.
(PDF)
