# Supplementary material for: The clinical significance of single or double bands in cerebrospinal fluid isoelectric focusing. A retrospective study and systematic review
Source: PLoS One. 2019 Apr 15;14(4):e0215410. doi: 10.1371/journal.pone.0215410 (PMC6464233; doi:10.1371/journal.pone.0215410)
Supplement: S1 Table — AIDS, acquired immune deficiency syndrome; CNS, central nervous system; St.p., status post. (PDF) [file pone.0215410.s002.pdf]

S1 Table. Clinical diagnoses of all patients with borderline OCB pattern

Diagnosis	No. of patients	No. of samples
**Inflammatory neurological disease (IND)**		
Multiple sclerosis	10	11
Meningitis	8	
Vasculitis	6	
Myelitis	5	7
Meningoencephalitis	4	5
HIV encephalopathy	3	
Parainfectious viral polyradiculomyelitis	2	3
Optic neuritis	2	
St. p. CNS tuberculosis	2	
Neuromyelitis optica	1	2
Acute disseminated encephalomyelitis	1	
Encephalitis	1	
Brain abscess	1	
Neurolues	1	
Hemichorea Sydenham	1	
Schilder's disease	1	
**Peripheral inflammatory neurological disease (PIND)**		
Guillain-Barré syndrome	17	21
Chronic inflammatory demyelinating polyneuropathy	4	7
Vestibular neuritis	5	
Ramsay-Hunt syndrome	2	
Neuralgic amyotrophy	1	
**Non-inflammatory neurological disease (NIND)**		
Stroke	18	
Polyneuropathy	14	15
Traumatic brain injury	5	
Amyotrophic lateral sclerosis	5	
Alzheimer's dementia	4	

(Spino)cerebellar ataxia	4	
Hydrocephalus	3	
CNS neoplasia	3	
Myelopathy	3	
Subcortical arteriosclerotic encephalopathy	3	
Metabolic-toxic encephalopathy	3	
Parkinson disease	2	
Dystonia	2	
Ischemic cranial nerve palsy	2	
Spastic spinal paralysis	2	
Leukoencephalopathy, toxic/ vascular	2	
Intracranial hypertension	1	2
Corticobasal degeneration	1	
Epilepsy	1	
Delir	1	
Developmental retardation	1	
Hypovitaminosis B12	1	
Myasthenia gravis	1	
Myopathy	1	
Meralgia paraesthetica	1	
Toxic opticopathy	1	
Traumatic peripheral nerve lesion	1	
Vascular malformation	1	
**Symptomatic controls (SC)**		
Vertebrogenic syndrome	20	
Bell's facial palsy	8	
Sensory disturbance/ dizziness	7	
Migraine	7	
Tension headache	4	
Thunderclap headache	1	
Other headache: associated with infection/ hypertension	4	
Benign paroxysmal position vertigo	1	

No neurological disease (NND)		
Somatoform disorder	7	8
Depression/ schizophrenia/ Bulimia nervosa/ panic attacks	5	
Lues latens	3	
Anterior ischemic optic neuropathy	3	
AIDS (defined by pneumocystis carinii pneumonia)	1	
Zoster ophtalmicus	1	

AIDS, acquired immune deficiency syndrome; CNS, central nervous system; St.p., status post
