# Supplementary material for: The clinical significance of single or double bands in cerebrospinal fluid isoelectric focusing. A retrospective study and systematic review
Source: PLoS One. 2019 Apr 15;14(4):e0215410. doi: 10.1371/journal.pone.0215410 (PMC6464233; doi:10.1371/journal.pone.0215410)
Supplement: S2 Table — In the whole cohort (n = 253), frequency of original OCB sub-pattern a, b and c are shown for each disease group (sub-pattern with the highest frequency are marked bold). Abbreviations: IND, inflammatory neurological disease; NIND, non-inflammatory neurological disease; NND, no neurological disease; OCB, oligoclonal bands; PIND, peripheral inflammatory neurological disease; SC, symptomatic control. (PDF) [file pone.0215410.s003.pdf]

S2 Table. OCB sub-pattern of all patients according to disease groups

	IND		NIND		PIND		SC		NND	
**Type *a***	23	41.8%	26	29.2%	10	27.8%	18	34.6%	4	19.0%
**Type *b***	6	10.9%	19	21.3%	5	13.9%	9	17.3%	2	9.5%
**Type *c***	26	**47.3%**	44	**49.4%**	21	**58.3%**	25	**48.1%**	15	**71.4%**

In the whole cohort (n=253), frequency of original OCB sub-pattern *a*, *b* and *c* are shown for each disease group (sub-pattern with the highest frequency are marked bold).

Abbreviations: IND, inflammatory neurological disease; NIND, non-inflammatory neurological disease; NND, no neurological disease; OCB, oligoclonal bands; PIND, peripheral inflammatory neurological disease; SC, symptomatic control
