# Supplementary material for: The clinical significance of single or double bands in cerebrospinal fluid isoelectric focusing. A retrospective study and systematic review
Source: PLoS One. 2019 Apr 15;14(4):e0215410. doi: 10.1371/journal.pone.0215410 (PMC6464233; doi:10.1371/journal.pone.0215410)
Supplement: S3 Table — Quartiles of IgG index were separated by values of 0.44, 0.50 and 0.56. IgG index was calculated as (CSF IgG/ serum IgG)/ (CSF albumin/ serum albumin). Abbreviations: IEF, isoelectric focusing; IgG, immunoglobulin G; OCB, oligoclonal bands; Q, quartile. (PDF) [file pone.0215410.s004.pdf]

S3 Table. Association of IgG index with reproducibility of borderline OCB pattern

IgG index	IEF I<br>Frequency of OCB borderline pattern<br>(n=234)						IEF II<br>Failure to reproduce identical OCB sub-pattern<br>(n=94)				IEF II<br>Failure to reproduce any borderline OCB pattern<br>(n=94)			
	Type *a*		Type *b*		Type *c*		Yes		No		Yes		No	
**Q1**	22	38.6%	6	10.5%	29	50.9%	15	75.0%	5	25.0%	13	65.0%	7	35.0%
**Q2**	18	30.5%	11	18.6%	30	50.8%	26	100.0%	0	0.0%	20	76.9%	6	23.1%
**Q3**	19	32.2%	12	20.3%	28	47.5%	27	90.0%	3	10.0%	23	76.7%	7	23.3%
**Q4**	18	30.5%	8	13.6%	33	55.9%	16	88.9%	2	11.1%	12	66.7%	6	33.3%

Quartiles of IgG index were separated by values of 0.44, 0.50 and 0.56. IgG index was calculated as (CSF IgG/ serum IgG)/ (CSF albumin/ serum albumin). *Abbreviations*: IEF, isoelectric focusing; IgG, immunoglobulin G; OCB, oligoclonal bands; Q, quartile
