# Supplementary material for: The clinical significance of single or double bands in cerebrospinal fluid isoelectric focusing. A retrospective study and systematic review
Source: PLoS One. 2019 Apr 15;14(4):e0215410. doi: 10.1371/journal.pone.0215410 (PMC6464233; doi:10.1371/journal.pone.0215410)
Supplement: S4 Table — (1) Studies that included only patients with NIND and SC are shown and used for specificity calculation. (2) Studies that included patients with NIND/SC and IND/PIND are shown. The N of patients with IND/PIND are substracted from the total N of controls, but not from the N of OCB negative patients. This approach results in a "Best OCB" scenario, where excluded IND/PIND are considered OCB positive. (3) Studies that included patients with NIND/SC and IND/PIND are shown. The N of patients with IND/PIND are substracted from the total N of controls and from the N of OCB negative patients. This approach results in a "Worst OCB" scenario, where excluded IND/PIND are considered OCB negative. (4) Studies from (1) and (2) are combined. (5) Studies from (1) and (3) are combined. Abbrevations: IND, inflammatory neurological disease; N, number; OCB, oligoclonal bands; PIND, peripheral inflammatory neurological diseae; SC, symptomatic control. (PDF) [file pone.0215410.s005.pdf]

S4 Table. Calculation of OCB diagnostic specificity using a cut-off ≥2 CSF-restricted bands

**Publication**	**ALL**			**NIND and SC (1)**		
	**Total N of controls**	**N of OCB negative patients**	**Specificity (%)**	**Total N of controls**	**N of OCB negative patients**	**Specificity (%)**
Bayart 2018	39	39	100	39	39	100
Christiansen 2018	97	91	94			
Gurtner 2018	144	121	84	144	121	84
Dias-Carneiro 2016	26	25	96	26	25	96
Zeman 2015	94	83	88	94	83	88
Abraira 2011	29	28	97	29	28	97
Gama 2009	19	19	100	19	19	100
Mygland 2007	255	245	96	255	245	96
Sa 2005	173	167	97	173	167	97
Bednarova 2005	15	14	93			
Villar 2005	334	332	99			
Haghighi 2000	50	48	96	50	48	96
Marchetti 1999	22	20	91	22	20	91
Cowdrey 1993	144	142	99			
Öhman 1992	211	207	98	211	207	98
Kostulas 1987	897	813	91	897	813	91
Link 1983	902	780	86			
**Total**	3451	3174	**92**	1959	1815	**93**

(1) Studies that included only patients with NIND and SC are shown and used for specificity calculation.

Publication	Total N of IND and PIND	Studies with mixed groups (2) IND and PIND exluded - Best OCB Case			Studies with mixed groups (3) IND and PIND exluded - Worst OCB Case		
		Total N of controls	N of OCB negative patients	Specificity (%)	Total N of controls	N of OCB negative patients	Specificity (%)
Bayart 2018							
Christiansen 2018	3	94	91	97	94	88	94
Gurtner 2018							
Dias-Carneiro 2016							
Zeman 2015							
Abraira 2011							
Gama 2009							
Mygland 2007							
Sa 2005							
Bednarova 2005	7	8	8	100	8	7	88
Villar 2005	63	271	271	100	271	269	99
Haghighi 2000							
Marchetti 1999							
Cowdrey 1993	9	135	135	100	135	133	99
Öhman 1992							
Kostulas 1987							
Link 1983	14	888	780	88	888	766	86
**Total**	96	1396	1285	92	1396	1263	90

(2) Studies that included patients with NIND/SC and IND/PIND are shown. The N of patients with IND/PIND are substracted from the total N of controls, but not from the N of OCB negative patients. This approach results in a "Best OCB" scenario, where excluded IND/PIND are considered OCB positive.
(3) Studies that included patients with NIND/SC and IND/PIND are shown. The N of patients with IND/PIND are substracted from the total N of controls and from the N of OCB negative patients. This approach results in a "Worst OCB" scenario, where excluded IND/PIND are considered OCB negative.

Publication	ALL STUDIES (4)<br>IND and PIND exluded - Best OCB Case			ALL STUDIES (5)<br>IND and PIND exluded - Worst OCB Case		
	Total N of controls	N of OCB negative patients	Specificity (%)	Total N of controls	N of OCB negative patients	Specificity (%)
Bayart 2018	39	39	100	39	39	100
Christiansen 2018	94	91	97	94	88	94
Gurtner 2018	144	121	84	144	121	84
Dias-Carneiro 2016	26	25	96	26	25	96
Zeman 2015	94	83	88	94	83	88
Abraira 2011	29	28	97	29	28	97
Gama 2009	19	19	100	19	19	100
Mygland 2007	255	245	96	255	245	96
Sa 2005	173	167	97	173	167	97
Bednarova 2005	8	8	100	8	7	88
Villar 2005	271	271	100	271	269	99
Haghighi 2000	50	48	96	50	48	96
Marchetti 1999	22	20	91	22	20	91
Cowdrey 1993	135	135	100	135	133	99
Öhman 1992	211	207	98	211	207	98
Kostulas 1987	897	813	91	897	813	91
Link 1983	888	780	88	888	766	86
**Total**	3355	3100	92	3355	3078	92

(4) Studies from (1) and (2) are combined.
(5) Studies from (1) and (3) are combined.

Abbrevations: IND, inflammatory neurological disease; N, number; OCB, oligoclonal bands; PIND, peripheral inflammatory neurological diseae; SC, symptomatic control
