# Supplementary material for: The clinical significance of single or double bands in cerebrospinal fluid isoelectric focusing. A retrospective study and systematic review
Source: PLoS One. 2019 Apr 15;14(4):e0215410. doi: 10.1371/journal.pone.0215410 (PMC6464233; doi:10.1371/journal.pone.0215410)
Supplement: S5 Table — Abbreviations: CSF, cerebrospinal fluid; N, number; OCB, oligoclonal bands. (PDF) [file pone.0215410.s006.pdf]

S5 Table. Calculation of OCB diagnostic specificity using a cut-off ≥3 CSF-restricted bands

**Publication**	**Total N of controls**	**N of OCB negative patients**	**Specificity (%)**
Gurtner 2018	144	127	88
Hegen 2016	161	161	100
Öhman 1992	211	207	98
McLean 1990	486	477	98
**Total**	1002	972	**97**

Abbreviations: CSF, cerebrospinal fluid; N, number; OCB, oligoclonal bands
